# Metabolic and anthropometric parameters contribute to ART-mediated CD4^+ ^T cell recovery in HIV-1-infected individuals: an observational study

**DOI:** 10.1186/1758-2652-14-37

**Published:** 2011-07-29

**Authors:** Livio Azzoni, Andrea S Foulkes, Cynthia Firnhaber, Xiangfan Yin, Nigel J Crowther, Deborah Glencross, Denise Lawrie, Wendy Stevens, Emmanouil Papasavvas, Ian Sanne, Luis J Montaner

**Affiliations:** 1 HIV-1 Immunopathogenesis Laboratory, the Wistar Institute, Philadelphia, PA, USA; 2 School of Public Health and Health Sciences, University of Massachusetts, Amherst, USA; 3 Clinical HIV Research Unit, University of the Witwatersrand, Johannesburg, South Africa; 4 Department of Chemical Pathology, National Health Laboratory Service and University of the Witwatersrand, Johannesburg, South Africa; 5 Department of Hematology and Molecular Medicine, National Health Laboratory Service and University of the Witwatersrand, Johannesburg, South Africa

## Abstract

**Background:**

The degree of immune reconstitution achieved in response to suppressive ART is associated with baseline individual characteristics, such as pre-treatment CD4 count, levels of viral replication, cellular activation, choice of treatment regimen and gender. However, the combined effect of these variables on long-term CD4 recovery remains elusive, and no single variable predicts treatment response. We sought to determine if adiposity and molecules associated with lipid metabolism may affect the response to ART and the degree of subsequent immune reconstitution, and to assess their ability to predict CD4 recovery.

**Methods:**

We studied a cohort of 69 (48 females and 21 males) HIV-infected, treatment-naïve South African subjects initiating antiretroviral treatment (d4T, 3Tc and lopinavir/ritonavir). We collected information at baseline and six months after viral suppression, assessing anthropometric parameters, dual energy X-ray absorptiometry and magnetic resonance imaging scans, serum-based clinical laboratory tests and whole blood-based flow cytometry, and determined their role in predicting the increase in CD4 count in response to ART.

**Results:**

We present evidence that baseline CD4^+ ^T cell count, viral load, CD8^+ ^T cell activation (CD95 expression) and metabolic and anthropometric parameters linked to adiposity (LDL/HDL cholesterol ratio and waist/hip ratio) significantly contribute to variability in the extent of CD4 reconstitution (ΔCD4) after six months of continuous ART.

**Conclusions:**

Our final model accounts for 44% of the variability in CD4^+ ^T cell recovery in virally suppressed individuals, representing a workable predictive model of immune reconstitution.

## Background

Chronic HIV infection is characterized by progressive loss of CD4^+ ^T cells; suppression of viral replication with antiretroviral agents results in most subjects in rapid CD4 recovery [1] and decreased T cell activation (e.g., CD38 expression [2]). Defective early recovery has been demonstrated to be associated with increased morbidity [3]; however, the extent of this recovery over time is difficult to predict, as it likely depends on multiple factors.

Baseline CD4+ T cell count remains the most relevant predictor of clinical progression and survival in subjects on antiretroviral therapy (ART) [4-8], but by itself it has been shown to inadequately account for the variability in ART-mediated immune restoration, and "on treatment" assessment of CD4+ T cells retains a better prognostic value [9]. Other factors positively associated with CD4+ T cell immune reconstitution include the presence of specific genotypes, such as Δ_32_CCR5 [10], antiretroviral regimen [11] and, in some studies, pre-ART viral load [12].

Immune activation of the T cell compartment (e.g., CD8^+ ^T cells), alterations of memory T cell subsets and depletion of innate immune subsets (e.g., NK and dendritic cells) are associated with advanced HIV infection [1,13-17]; however, while most of these cell subsets are at least partially recovered on ART, even though with different kinetics, their potential association with early CD4 recovery has not been explored.

In addition to viral and immunologic parameters, metabolic factors have been shown to be associated with disease progression, and are putative candidates to predict CD4 recovery: advanced HIV infection (i.e., low CD4 counts) is associated with chronic inflammation and increased immune activation, with alteration of metabolic parameters associated with lipid metabolism and increased atherogenic risk (as assessed by increased carotid intima-media thickness) in subjects of both sexes [18,19]. A number of studies have reported that subjects with advanced HIV infection have lower high-density lipoprotein (HDL) cholesterol, higher low-density lipoprotein (LDL) cholesterol and triglycerides [20,21], and CD4 counts appear to directly correlate with HDL cholesterol [22,23].

The existence of a relationship between metabolic markers, viremia and immune activation is also suggested by the observation that ART-mediated suppression of HIV replication results in a rapid normalization of a number of markers linked to cardiovascular risk [24].

While these observations highlight the negative effects of HIV infection on lipid metabolism and overall atherogenic risk, it is of note that cohort-based observations indicate that high adiposity (which is normally associated with insulin resistance, dyslipidemia and atherogenesis) might be beneficial for HIV-infected individuals, contributing to lower steady state viral replication and slower disease progression [25,26]. Altogether, these observations suggest that adipose tissue accumulation and distribution may affect the immunological host/virus equilibrium in chronic HIV infection; however, the impact of adiposity on ART-mediated immune reconstitution remains undefined.

In a reported multivariate analysis, subject age, nadir and baseline CD4 count and initial viral load were found to be inversely associated with early CD4 response to suppressive ART [12]; importantly, the predictive value of subject gender was ascribed to its effect on baseline CD4 measurements [12,27]. Predictive logistic regression models for incomplete CD4 response have been developed, based on subject age, baseline CD4^+ ^T cell count and early CD4 response [28]; however, to our knowledge, there are at present no satisfactory models that adequately predict early (less than six months) CD4^+ ^T cell immune reconstitution. To our knowledge, adiposity-associated metabolic markers (e.g., BMI, serum lipid fractions, HOMA-2), have not used in these models, and their predictive role remains unclear.

Based on the reported association of viremia and CD4 counts with body mass index (BMI) and serum lipid levels, we sought to determine: (1) if adiposity and markers associated with lipid metabolism can affect the degree of early (six months [3]) immune reconstitution after ART; and (2) if metabolic parameters could contribute to a predictive model for immune reconstitution that includes pre-ART viral, immune activation and CD4^+ ^T cell counts. The present study followed a cohort of ART-naïve, HIV-infected South African subjects. We demonstrate that metabolic parameters measured before ART have a significant effect on the degree of immune reconstitution attained after six months of continuous ART and contribute significantly to a predictive model of CD4^+ ^T cell immune reconstitution.

## Methods

### Study subjects

We assessed 69 ART-naïve, HIV-infected subjects initiating ART (d4T, 3TC and lopinavir/ritonavir) at the Clinical HIV Research Unit of the Themba Lethu Clinic, Johannesburg, South Africa (21 males, 48 females). Medical history was obtained from the clinic record and by interview. Written informed consent was obtained from all participants as per University of the Witwatersrand Ethics Committee- and Wistar Institute Institutional Review Board-approved study protocol.

### Adiposity measurements

Baseline height, weight and anthropometric measurements were obtained pre-ART by trained study personnel; BMI was calculated as weight (kg) divided by height (m)^2^. Dual energy X-ray absorptiometry (DEXA) scans were performed using a Hologic QDR-2000 scanner, assessing limb and trunk fat and lean mass. Magnetic resonance imaging (MRI) scans were performed using a Toshiba Flexart 0.5 T; a single L4-L5 axial section was used to determine sagittal diameter, visceral, subcutaneous, total abdominal and peri-renal fat. The analysis was conducted using V3.51*R553 software.

### Clinical laboratory testing

CD4 counts were assessed at baseline (CD4BL, last available measurement prior to ART initiation) and approximately 36 weeks from ART initiation (range 220-259 days; CD4_END_), using the single platform method described by Scott and Glencross [29]. Serum from fasting blood draws was tested for HDL cholesterol, triglycerides and glucose using a Roche Integra analyzer 400 (Roche Diagnostics, Mannheim, Germany); LDL cholesterol was estimated using the Friedewald formula [30]. HIV-1 infection was confirmed via rapid antibody testing and/or ultra-sensitive PCR, (Roche COBAS Ampliprep/COBAS Amplicor v1.5 methods), with viral load suppression to < 50 copies/ml on ART confirmed every eight weeks.

### Immunology measurements

Four-colour flow cytometry stainings to assess immunological parameters were performed on whole blood using custom-made lyoplates (BD Biosciences, Palo Alto, CA). The following antibody combinations were used for the specified target populations: T cell activation/differentiation: CD8, CD28, CD3, CD38; and T cell activation: CD8, CD95, CD3, HLA-DR. After RBC lysis, sample fluorescence data were acquired with a FACScalibur flow cytometer and analyzed using CellQuest software (BD Biosciences). Isotype-matched control antibodies were used as negative controls for gate positioning.

### Statistical analysis

Summary statistics (mean, standard deviation, median, min and max) are reported for each independent variable (listed in Table 1) at baseline. Simple linear regression models were fitted to the primary endpoint ΔCD4 (ΔCD4 = endpoint CD4 count - baseline CD4 count). Multivariable models were generated using an iterative, stepwise model building procedure, combining forward and backward selection [31]. Differences in time to suppression by BMI category were assessed using a Kaplan Meier test. All statistical tests were performed using R vers. 2.10.0 [32].

**Table 1:** Baseline (pre-ART) cohort characteristics

Variable	25th quantile	Median	75th quantile	Mean	Standard deviation
Gender (female/male ratio)	2.29 (48/21)				
Age (years)	29.0	33.0	39.0	34.6	8.2
Baseline CD4 count (cells/mm^3^)	221.0	243.0	292.0	259.8	61.6
Baseline log_10 _VL	4.0	4.7	5.1	4.5	0.8
Total fat mass (DEXA, g)	9356.1	19451.7	28589.5	20719.7	11801.5
Total lean mass (DEXA, g)	39458.8	42455.1	48867.2	43582.5	6038.0
Fat ratio (DEXA, %)	16.2	32.7	39.5	29.6	12.6
Total abdominal fat (MRI, cm^2^)	144.0	294.7	414.6	311.3	191.3
Cholesterol (mmol/L)	3.1	3.5	4	3.6	0.8
HDL-associated cholesterol (mmol/L)	0.9	1.1	1.3	1.1	0.3
LDL-associated cholesterol (mmol/L)	1.6	2.1	2.5	2.1	0.7
Triglycerides (mmol/L)	0.6	0.8	1	0.8	0.3
LDL/HDL cholesterol ratio	1.5	1.8	2.6	2.3	2.7
Waist circumference (cm)	73.0	78.5	87.5	80.9	11.3
Waist/hip ratio	0.7	0.8	0.8	0.8	0.1
Fasting glucose (mmol/l)	4.0	4.2	4.4	4.3	0.6
BMI (kg/m^2^)	24.5	26.8	29.9	28.1	5.1
CD95^+ ^CD8^+ ^T cells (%)	81.9	89.9	95.9	85.7	14.6

## Results

### Cohort characteristics

The baseline characteristics of our cohort are summarized in Table 1. The median baseline CD4 count (CD4_BL_) was 243 cells/mm^3^, with a median log_10_VL (log_10_VL_BL_) of 4.7. Median BMI was 26.8kg/m^2^, with 70% of the cohort being overweight or obese (48 of 69 subjects with BMI > 25); median LDL/HDL ratio was 1.8, and median serum fasting glucose was 4.2 mmol/l. According to the Adult Treatment Panel III guidelines [33], 65% of the subjects (45 of 69) had low HDL cholesterol levels [61% < 1mM (male) or < 1.3 mM (female)], 3% of the subjects had elevated triglycerides (≥ 1.7 mM), 3% had elevated total cholesterol (≥ 5.0 mM), and 7% had elevated LDL cholesterol (≥ 3.0 mM).

After 24 weeks of ART, the median endpoint CD4 count (CD4_END_) was 421 cells/mm^3 ^(interquartile range: 355-505), with a median gain (ΔCD4) of 172 (IQR 92-247) CD4^+ ^T cells; five subjects (5.2%) failed to gain CD4 on ART in the presence of viral suppression (immunological failure). As expected, the spread of the distribution in CD4 gain after ART supports the hypothesis that, in addition to viral suppression alone, other factors may determine the extent of immune reconstitution on ART.

### Baseline CD4 count, viral load and cellular activation affect immune reconstitution in response to ART

The unadjusted effects of baseline characteristics on ART-mediated immune reconstitution, as measured by ΔCD4 count, are summarized in Table 2. As expected, the effect of log_10_VL_BL _on ΔCD4 was observed to be positive (effect estimate 56.0, corresponding to an increase of 56 CD4^+ ^T cells/mm^3 ^in ΔCD4 per log of VL; p = 0.002; adjusted R^2 ^= 0.12), suggesting that subjects with high levels of viral replication had the most benefit from pharmacological suppression in terms of CD4 recovery. Conversely, lower baseline CD4_BL _correlated with higher ΔCD4 (effect estimate -0.61, corresponding to a decrease of 0.61 CD4^+ ^T cells/mm^3 ^in ΔCD4 per unit of CD4_BL_; p = 0.008; R^2 ^0.08), indicating a greater benefit of therapy in these subjects.

**Table 2:** Association of baseline variables with ΔCD4: model fitting with single variables

Predictor	Estimate	**S.E**.	Pr(> |t|)	Adjusted R^2^
Age	-2.773	1.751	0.1180	0.0217
Sex	-26.283	31.231	0.4030	-0.0043
CD4_BL_	-0.607	0.224	0.0085	0.0854
Log_10_VL	56.048	17.110	0.0017	0.1252
Total fat mass (DEXA)	0.000	0.001	0.8935	-0.0147
Total lean mass (DEXA)	-0.002	0.002	0.3068	0.0009
Total fat % (DEXA)	0.745	1.148	0.5184	-0.0086
Total abdominal fat (MRI)	-0.007	0.076	0.9293	-0.0148
LDL/HDL ratio	-9.432	5.358	0.0829	0.0299
Waist circumference	-1.128	1.281	0.3817	-0.0033
Waist/hip ratio	-458.084	183.071	0.0148	0.0718
Fasting glucose	-28.171	23.307	0.2310	0.0067
BMI	-0.962	2.828	0.7348	-0.0132
CD95^+ ^CD8^+ ^T cells	3.136	0.919	0.0011	0.1354

Baseline levels of CD95^+ ^CD8^+ ^T cells, an immune activation parameter previously shown to predict pDC recovery on ART [34], had a significant positive effect on ΔCD4 (Table 2; effect estimate 3.14, p = 0.001), and had a predictive association with CD4 (adj. R^2 ^= 0.13). We did not detect a significant association of CD38 or HLA-DR expression on CD4^+ ^or CD8^+ ^T cells with CD4 outcomes (not shown).

### Effect of metabolic and anthropometric parameters on immune reconstitution outcomes

As summarized in Table 2 a meaningful negative association with ΔCD4 was observed for waist/hip ratio (effect estimate -458.1, p = 0.015, adjusted R^2 ^= 0.072); no association was observed for BMI or gender, suggesting that the relationship is limited to central adiposity, as assessed by waist/hip ratio. LDL/HDL cholesterol ratio (effect estimate -9.432, p = 0.083, adjusted R^2 ^= 0.03) was also associated with ΔCD4, unlike other lipid measures (not shown).

**Figure 1:**
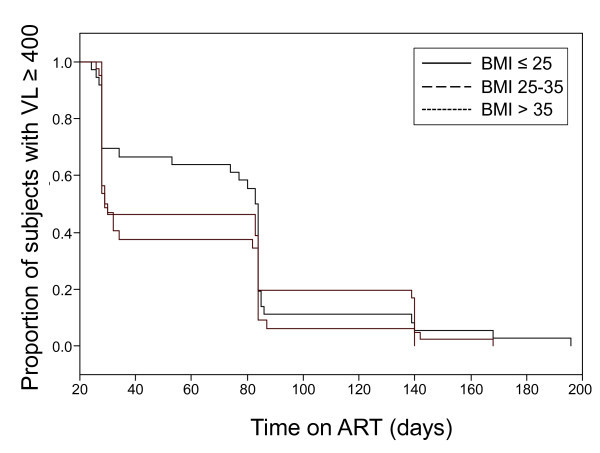
**Effect of BMI on the time to ART-mediated suppression**. The proportion (%) of viremic subjects was assessed at each study visit for six months following ART initiation. Kaplan-Meier curves are displayed for normal/underweight (BMI < 25 kg/m^2^; n = 21; continuous line), overweight (BMI 25-30 kg/m^2^; n = 31; dashed line) and obese (BMI > 30 kg/m^2^; n = 17; dotted line). Differences between curves are not significant.

To assess if the observed negative effect of central adiposity (i.e., waist/hip ratio) and lipid indicators could be associated with incomplete or delayed suppression of viral load below 50 copies/ml, we compared the proportion of individuals achieving viral suppression (VL < 400 c/ml) over time between normal/underweight, overweight and obese subjects, using a Kaplan-Meier analysis. The survival curves were not significantly different (Figure 1). In addition, we could not detect an association between BMI or waist/hip ratio and time to suppression (not shown). Thus, our data do not support the conclusion that the negative effect of central adiposity on CD4 immune reconstitution observed in this cohort is caused by differences in rates of virological suppression.

### Multivariable analysis of predictors of CD4 recovery on ART

**Figure 2:**
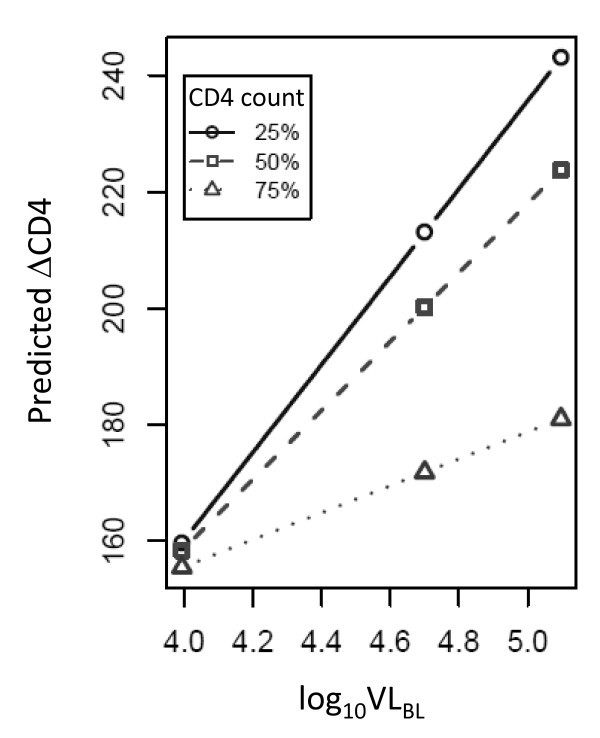
**Mixed effect modelling of the effect of baseline CD4 percentile and viral load on CD4+ T cell reconstitution**. The complete model (Table 3) was fitted to the data: linear predicted ΔCD4 as a function of log_10_VL is plotted for baseline CD4 count = 25^th ^quantile (circles), 50 quantile (squares) and 75 quantile (triangles) of the baseline CD4 distribution.

We used a multivariable approach to estimate the combined effect of multiple baseline variables on CD4 recovery on ART. The adjusted R^2 ^of each model tested is reported in Table 3; together, CD4_BL _and log_10_VL_BL _accounted for approximately 18% of the variability in ΔCD4 (adj. R^2 ^= 0.1828). We also observed a significant interaction between CD4_BL _and log_10_VL_BL _(Figure 2), indicating that the effect of an increase in log_10_VL_BL_on ΔCD4 was greater among individuals with lower CD4_BL _than among individuals with higher CD4_BL_; modelling this interaction improved the model predictivity to approximately 22% (adj. R^2 ^= 0.219). As CD8^+ ^T cell activation has been associated with clinical outcomes in past studies, we tested whether including in this model the frequency of CD95^+ ^CD8^+ ^T cells, the only activation term individually associated with the ΔCD4 outcome, would improve the predictivity of CD4_BL _and VL_BL_: our results indicate an adj. R^2 ^of 0.2751 for the combined model, supporting the use of an activation term.

**Table 3:** Adjusted R^2 ^for linear models of ΔCD4

Variable(s) included as predictors	Adjusted R^2^	-2 log ^L
CD4_BL_	0.0854	847.28
log_10_VL	0.1252	844.20
CD4_BL _+ log_10_VL	0.1828	838.47
CD4_BL _+ log_10_VL + (CD4_BL _× Log_10_VL)^a^	0.2190	834.29
CD4_BL _+ log_10_VL + (CD4_BL _× Log_10_VL) + Waist/hip ratio	0.2453	830.85
CD4_BL _+ log_10_VL + (CD4_BL _× Log_10_VL) + LDL/HDL ratio	0.3380	828.08
CD4_BL _+ log_10_VL + (CD4_BL _× Log_10_VL) + CD8^+^CD95^+ ^T cells	0.2751	821.81
CD4_BL _+ log_10_VL + (CD4_BL _× Log_10_VL) + LDL/HDL ratio + Waist/hip ratio	0.3673	817.60
CD4_BL _+ log_10_VL + (CD4_BL _× Log_10_VL) + LDL/HDL ratio + Waist/hip ratio + CD8^+^CD95^+ ^T cells	0.4377	808.36

a: interaction term

The metabolic terms, LDL/HDL cholesterol ratio and waist/hip ratio, together accounted for 11% of ΔCD4 variability (adj. R^2 ^= 0.1122, similar to CD4_BL _alone); when both metabolic parameters were added to CD4_BL _and VL_BL_, the model accounted for almost 37% of ΔCD4 variability (adj. R^2 ^= 0.3673), confirming the role of these metabolic terms as outcome predictors.

The final model, selected for best fit by assessing the models' -2 log likelihood (see Table 4) included CD4_BL_, log_10_VL_BL_, LDL/HDL ratio, waist/hip ratio and CD95^+ ^CD8^+ ^T cells, in addition to an interaction term between CD4_BL _and log_10_VL_BL_: all of the variables selected had a significant independent effect on the ΔCD4; the interaction CD4_BL _and log_10_VL_BL _also remained significant. This model accounted for almost 44% of the variability in ΔCD4 (R^2 ^= 0.4377), which is approximately twice as much as the best performing CD4_BL _and log_10_VL_BL_-based model, and 1.6 times greater than the model including CD4_BL_, log_10_VL_BL _and CD95 expression. The addition of an interaction term between CD4_BL _and CD95^+ ^CD8^+ ^T cells resulted in a further increase of the model predictivity (adj. R^2 ^= 0.46, not shown), but as the effect of the interaction term *per se *was not significant (p = 0.057), it was not included in the final model.

**Table 4:** Multivariable analysis: complete model parameter estimates

Coefficient	Estimate	Standard error	p
Intercept	-721.3331	372.7459	0.0575
CD4_BL_	2.8829	1.2345	0.0228
log_10_VL_BL_	238.3317	72.7549	0.0017
CD4_BL _× log_10_VL_BL_	-0.7369	0.2753	0.0095
LDL/HDL ratio	-17.3449	4.2669	0.0001
Waist/hip ratio	-294.0370	146.6771	0.0494
CD95^+ ^CD8^+ ^T cells	2.3330	0.7827	0.0041

Adjusted R^2 ^= 0.4377

## Discussion

We report that a multivariable model using pre-ART viral load, immunological parameters and metabolic variables predicts short-term CD4 recovery in subjects initiating ART to a substantially higher degree than previously reported models. The variability of the extent of immune reconstitution levels (i.e., CD4 gain) in response to ART-mediated viral suppression, confirmed in our cohort, suggests that a number of factors, in addition to successful viral suppression, might affect the extent of immune recovery. Pre-treatment CD4 counts, viral load and immune activation are recognized to play a role in determining the levels of immune recovery [8,10,12,34-36], but individually they have limited usefulness as predictors of early CD4 recovery [9]. All individuals in our cohort received the same ART regimen, thus ruling out effects of post-ART CD4 recovery linked to differences in treatment regimens, as observed in other studies [11].

Our results confirm that pre-ART VL, CD4 count and cellular activation (i.e., CD95 expression [37,38]), alone or in combination, have a significant, but limited value in predicting the CD4^+ ^T cell recovery outcome, explaining only 21% of its variability. The effect of baseline CD4 on ΔCD4 was negative, confirming a prior report [39]; unlike earlier studies [8], we did not assess the effect of baseline CD4^+ ^T cell levels on CD4 immune reconstitution, which was found to be positive, as we considered ΔCD4 (a measure incorporating CD4_BL_) more relevant to assessing an immune reconstitution response. Prior studies have reported an effect of age and gender on CD4 outcomes of treatment [12,27]; while we failed to detect such associations in our cohort, the difference in outcome measured (ΔCD4 vs. CD4 count at endpoint) is likely responsible for this discrepancy.

We found a meaningful negative association between LDL/HDL ratio and CD4^+ ^T cell recovery. While this finding is novel, associations of lipid levels and viral replication have been reported [40-43], suggesting the possibility that the observed relationship between LDL/HDL ratios and immune recovery may result in part from direct effects on viral function. A number of studies have demonstrated the effects of membrane cholesterol and lipid rafts on viral penetration and/or budding [44-46]. Moreover, apolipoprotein A1, a component of HDL, has been shown to directly affect the viral life cycle at the viral entry and syncytium formation stages [47-49]). A recent study indicated an association of hypocholesterolemia with a reduced response to ART [50], and studies with cholesterol-lowering agents have shown mixed results [51-56].

Adiposity has generally been associated with better viral control and slower disease progression in ART-naïve, HIV-positive subjects [25,26,57,58]. While in our cohort, BMI did not predict ΔCD4 in response to ART, in keeping with a prior report that did not detect a lack of response to ART in obese subjects [59], we did observe a negative association between waist/hip ratio and CD4 gain, indicating that subjects with low waist to hip ratios (i.e., with low central adiposity) are likely to have better immunologic recovery. One possible hypothesis to explain the disconnect between BMI and waist/hip ratio predictive values is that antiretroviral drugs may be metabolized differently or be less bio-available in subjects with higher central adiposity (i.e., high waist/hip ratio). It is also possible that abdominal adipose tissue, particularly the visceral depot, secretes factors that may modulate the effects of the ART or directly interfere with immune reconstitution [60].

While we did not evidence significant differences in time to viral suppression to < 50 c/ml between normal, overweight and obese subjects (Figure 1), we cannot exclude that metabolic events may be associated with residual levels of viral replication, affecting in turn short-term CD4 recovery. Importantly, the overall HDL- cholesterol values in our cohort were low, with 61% of the subjects being classified as dyslipidemic [33], in keeping with prior reports in HIV-infected African populations [61,62], and there was a high prevalence of overweight/obesity [63] (79% of women and 48% of men had BMI > 25 kg/m^2^). Based on these observations, as well as the present contribution, further studies in larger cohorts will be necessary to determine if metabolic parameters play the same role in low-central adiposity individuals, and to further explore the relationship between lipids and viral control.

Altogether our data indicate that metabolic parameters contribute to predicting the degree of immune reconstitution achieved upon viral suppression. While our study does not address the pathophysiologic mechanisms underlying this relationship, prior reports indicate that fat accumulation promotes low-level inflammation, which, in turn, has been shown to be associated with lack of immunologic reconstitution [38], suggesting a possible biological pathway.

By including pre-ART metabolic parameters in conjunction with baseline CD4, viral load and immune activation, our final model accounts for 44% of the variability in CD4^+ ^T cell gain in response to viral suppression, representing, to our knowledge, the best predictive model on immune reconstitution to date, and represents a marked improvement over more conventional assessments (e.g., baseline CD4^+ ^T cell counts alone or with viral load).

While not designed to support clinical interventions, our results, if supported by validation in a larger cohort, suggest the testable hypothesis that clinical and behavioural interventions aimed at reducing weight in subjects with central adiposity, as well as pharmacological intervention aimed at improving LDL/HDL ratios (e.g., statins), might improve the immunological outcomes or ART, at least in the short term.

As with all modeling techniques, there are limitations to our findings. In the first place, we modeled the effect of the assessed variables on the change in CD4 between baseline and six months on ART: it remains to be determined if incorporating multiple early CD4 measurements would improve the predictivity of the model. Moreover, the predictive value of the model will have to be validated in a larger independent cohort.

In addition, due to the relatively small size of the study, we did not assess the effect of clinical conditions that could affect some of the parameters studies here (e.g., hypertension, diabetes).

As we gain a more accurate estimate of response to ART, it remains to be determined, through further studies, how each variable impacts CD4 recovery mechanistically and whether additional predictors may improve the reliability of the prediction.

## Conclusions

We report for the first time that metabolic markers can contribute significantly to the variability of immune reconstitution outcomes following ART initiation in a cohort of HIV-1-infected South African subjects. While the current study clearly establishes the predictive potential for metabolic markers, further studies will be required to determine the cost effectiveness of this predictive approach, and to determine whether additional longitudinal measurement would further improve the model performance.

## Competing interests

The authors declare that they have no competing interests.

## Authors' contributions

LA was responsible for study design, data management, data analysis, and manuscript and illustration preparation. ASF supervised the statistical analysis, and contributed to data discussion and manuscript preparation. CF was responsible for clinical coordination and patient interaction, and contributed to data discussion and manuscript revision. XY was responsible for statistical analysis, and contributed to data discussion and manuscript revision. NJC was responsible for lipid assessment, and contributed to critical analysis, data discussion and manuscript preparation. DG was responsible for flow cytometry supervision, and contributed to data discussion and manuscript revision. DL was responsible for flow cytometry analysis and CD4 assessment, and contributed to manuscript revision. WS was responsible for clinical laboratory supervision, and contributed to data discussion and manuscript preparation. EP contributed to data discussion and manuscript revision. IS was responsible for supervising clinical coordination and patient interaction, and contributed to data discussion and manuscript preparation. LJM was responsible for supervising immunology laboratory assessments, and contributed to study design, critical analysis and manuscript preparation.

All authors read and approved the final manuscript.
